# Low-Dose Interleukin-2 Therapy: A Driver of an Imbalance between Immune Tolerance and Autoimmunity

**DOI:** 10.3390/ijms151018574

**Published:** 2014-10-15

**Authors:** Agata Kosmaczewska

**Affiliations:** Institute of Immunology and Experimental Therapy, Department of Experimental Therapy, Polish Academy of Sciences, 12 R. Weigla St., 53-114 Wroclaw, Poland; E-Mail: kosmacz@iitd.pan.wroc.pl; Tel.: +48-71-337-11-73 (ext. 273); Fax: +48-71-337-21-71

**Keywords:** interleukin-2, immunotherapy, immune-mediated disease, autoimmunity, immune tolerance, clinical trial

## Abstract

For many years, the role of interleukin-2 (IL-2) in autoimmune responses was established as a cytokine possessing strong pro-inflammatory activity. Studies of the past few years have changed our knowledge on IL-2 in autoimmune chronic inflammation, suggesting its protective role, when administered at low-doses. The disrupted balance between regulatory and effector T cells (Tregs and Teffs, respectively) is a characteristic of autoimmune diseases, and is dependent on homeostatic cytokines, including IL-2. Actually, inherent defects in the IL-2 signaling pathway and/or levels leading to Treg compromised function and numbers as well as Th17 expansion have been attributed to autoimmune disorders. In this review, we discuss the role of IL-2 in the pathogenesis of autoimmune diseases. In particular, we highlight the impact of the dysregulated IL-2 pathway on disruption of the Treg/Th17 balance, reversal of which appears to be a possible mechanism of the low-dose IL-2 treatment. The negative effects of IL-2 on the differentiation of follicular helper T cells (Tfh) and pathogenic Th17 cells, both of which contribute to autoimmunity, is emphasized in the paper as well. We also compare the current IL-2-based therapies of animal and human subjects with immune-mediated diseases aimed at boosting the Treg population, which is the most IL-2-dependent cell subset desirable for sufficient control of autoimmunity. New perspectives of therapeutic approaches focused on selective delivery of IL-2 to inflamed tissues, thus allowing local activity of IL-2 to be combined with its reduced systemic and pleiotropic toxicity, are also proposed in this paper.

## 1. Introduction

Autoimmune diseases, such as rheumatoid arthritis (RA), systemic lupus erythematosus (SLE), or type-1 diabetes (T1D), are examples of the lost tolerance to self antigens, in which one’s own immune system is able to destroy particular cells of the host. Autoimmunity is characterized by the broken balance between Treg and Teff cells. Functional cross-talk between Tregs and Teffs is essential for the maintenance of immune homeostasis and is fulfilled by homeostatic cytokines, including IL-2 [1]. In general, Tregs require IL-2 secreted by activated Teffs, while Teffs need Tregs to prevent excessive cell activation [2].

Anti-inflammatory activity of IL-2 in autoimmunity has been generally accepted, since inherent defects in the IL-2 pathway, decreased response to IL-2, and/or reduced IL-2 availability, resulting in the impairment of Treg function, frequency and/or survival, have been found in many autoimmune disorders both in mice [3,4,5,6,7] and humans [8,9]. In some of them, such as SLE and T1D, it could even be a characteristic of the disease [10,11,12,13,14,15]. Many studies, including ours, have shown that lowered IL-2 serum levels may even be a biomarker of progression of the disease [1,16]. As a rather protective cytokine, low-dose IL-2-based therapies may be used to restore suitable IL-2, and in consequence a beneficial Treg/Teff ratio, by shifting the balance from Th17-mediated inflammatory conditions to a Treg-mediated tolerant state in an animal model of lost tolerance [5,17,18,19,20,21,22] as well as in human clinical trials [9,23,24,25,26].

In this review, we discuss the role of IL-2 in pathogenesis of autoimmune diseases, particularly regarding its positive considerable impact on Treg cell fitness and suppressive function. We also report the significance of IL-2 in eliminating either the Tfh cells, which promote autoantibody production, or the pathogenic Th17 cells, both of which play a major role in the development of autoimmune diseases. Additionally, we present up-to-date preclinical and clinical studies on low-dose IL-2-based therapy of autoimmune disorders, and focus special attention on suitable dosing and frequency of IL-2 administration in the treatment of autoimmune diseases to avoid inappropriate immune responses. Finally, future perspectives for use of IL-2 in the treatment of autoimmune disease are also presented.

## 2. The Role of IL-2 (Interleukin-2) in the Pathogenesis of Autoimmune Diseases: The Positive Effects of IL-2 on Treg Fitness and Homeostasis

IL-2 appears to be the first molecule that is able specifically and directly to expand Tregs beneficial in the treatment of autoimmunity, suggesting its tremendous therapeutic potential [21,22,23,24,25]. IL-2 is primarily produced by activated T cells and dendritic cells (DCs) and its receptors are expressed on T cells; thereby these cells are effectors of IL-2. However, differential expression of IL-2 receptors on particular T cell subsets leads to selective IL-2-driven expansion of Tregs and activated Teffs, since both subsets express the high affinity IL-2 receptor alpha (IL-2Ralpha or CD25) that pairs with the low-affinity IL-2 receptor beta (IL-2Rbeta or CD122) and the common gamma chain. Therefore, selective expression of IL-2 receptors determines the magnitude and persistence of its response within the T cell compartment.

At present, it is widely accepted that IL-2 is able to play a critical role mainly in Treg fitness and homeostasis, partially due to the constitutive high expression of CD25 [5,7,8,27], which makes them a highly IL-2-dependent cell subset. In Tregs, IL-2 signaling is mainly transmitted by the signal transducer and activator of transcription (STAT) pathway, resulting in induced FOXP3 expression, peripheral expansion and inhibitory function of Tregs [28,29,30,31,32]. Actually, in the mouse model of autoimmunity, it has been observed that an absence of IL-2 resulted in insufficient Treg expansion, while it disturbed neither Teff expansion nor Teff responses to enhanced levels of the pro-inflammatory cytokine IL-15 [33]. Decreased function and/or numbers of Treg cells have been demonstrated in many autoimmune diseases [17,34,35,36,37,38], and in some cases, it was even correlated with disease severity [36,39]. In our recent study, we consistently reported a correlation between irreversible IL-2 deficit in peripheral blood (PB) of the most advanced RA and Treg cell down-regulation [16], and these findings were in line with others [40,41]. Remarkably, the Treg cell defects strongly promoting autoimmune responses appeared to be restored by exogenous IL-2 administered at low doses both in mice [20,21,42,43] and humans [23,24,25], contrasting with the lack of such responses in Teff cells. The doses of IL-2 used in the studies were as follows: mice were injected intraperitoneally (ip) with 2.5 × 10^4^ IU/day of recombinant human (rh) IL-2 [5,18,21,22] or 1–2.5 µg/day of recombinant mouse (rm) IL-2 [19,20]; and humans were administered subcutaneously (sc) with 0.33–4.5 × 10^6^ IU/day [9,23,24,25,26]. In our extended *in vitro* study [44], we accordingly observed that exogenous IL-2 at a physiologic dose of 500 IU/mL was able to correct Treg numbers and function compromised only in progressive RA patients with a baseline IL-2 systemic deficit, which confirms the strong dependence of Tregs on IL-2 availability. Also, IL-2 added to the culture at a dose of 100 IU/mL was able to selectively up-regulate FOXP3 expression in Tregs, but not in Teffs, consistently with the finding of the IL-2-mediated stronger induction of the Janus kinase (JAK)/STAT signaling than phosphatidylinositide 3-kinase (PI3K) signaling pathway [45].

The Treg dependence on suitable amounts of IL-2 has been primarily demonstrated in IL-2/IL-2R knock-out mice exhibiting severe autoimmunity leading to early death [46,47,48]. It is worth mentioning that in humans, alterations in IL-2/IL-2R signaling measured by the levels of STAT5 phosphorylation (pSTAT5) and/or reduced IL-2 availability, which have been clearly described and linked to type-1 diabetes (T1D) [48,49,50], result in the impairment of Treg function, but usually not in Treg frequency [34,49]. In fact, lower pSTAT5 levels in both Treg and CD4+ memory T cells have been observed over the course of T1D [51]. In consequence, a hallmark of the T cell compartment in T1D is impaired FOXP3 expression in Tregs [51] and higher susceptibility of Tregs to apoptosis as well as expansion of Th17 cells in IL-2 deprivation [8,52]. The diminished IL-2 response in T1D may result from genetic variation, including multiple T1D-associated variants identified in at least 4 genes encoding unique proteins involved in the IL-2 pathway, including IL-2, IL-2Ralpha, IL-2Rbeta, and PTPN2 (protein tyrosine phosphatase N2) genes [50,53,54,55]. Similarly, reduced IL-2 expression in NOD mice, an experimental model of T1D, was reported to contribute to qualitative rather than quantitative impairment of Treg cells. It has been shown that decreased IL-2 availability can lead to a transient increase in CD62L− Tregs, which were reported to have limited suppressor activity [56,57]. Those studies markedly showed that IL-2 is capable of affecting the balance between CD62L− and CD62L+ Tregs, thus regulating the inhibitory function of the Treg cell compartment. The consequence of Treg dysfunction associated with the lost tolerance is lower suppression of pathogenic Teffs including autoreactive T cells, thus allowing them to become hyperactivated during immune responses.

## 3. IL-2 in the Pathogenesis of Autoimmune Diseases: The Role of IL-2 in Eliminating Tfh and Th17 Cells

In addition to the positive effects of IL-2 on Tregs and the role of Tregs in suppressing immune responses, one should note that IL-2 can also promote Teff cell stimulation and differentiation depending on the transient CD25 expression. Depending on the environmental cytokines, CD4+ T cells differentiate into various types of T cells, namely follicular or non-follicular Teffs (Tfh or non-Tfh, respectively) to control antigen-specific immune responses [58]. Non-follicular Teffs include Th1, Th2, Th17, and induced Treg cells, whereas Tfh cells are a subset of CD4+ T cells migrating into B cell follicles in the lymphoid organ to provide specialized help to germinal center (GC) B cells [59,60]. Therefore, Tfh cells promote humoral immunity including autoantibody production [61]. Dysregulation of Tfh cell differentiation has a considerable impact on the T-dependent antibody responses, thus driving the production of autoantibodies that are pathogenic in several autoimmune diseases; recent studies have demonstrated that Tfh differentiation is positively correlated with intensity of the humoral response [60,61,62].

Positive regulation of Tfh differentiation has been well characterized, indicating a role of pro-inflammatory cytokines IL-6 and IL-21 as well as direct interactions with antigen-presenting cells. All these positive signals are capable of the induction of transcription factor B cell lymphoma-6 (Bcl-6) in Tfh cells [58]. At present, little is known about repressors of Tfh cells. One of the antagonists of Bcl-6 is the transcription factor Blimp-1 found at high levels in non-Tfh effector cells [62]. Since Bcl-6 and Blimp-1 are reciprocally regulated, this axis determines the direction of Teff differentiation toward Tfh or non-Tfh cells [58,62]. Therefore, negative regulation of Tfh differentiation could be achieved, at least in part, by inducing repressors of Bcl-6, such as Blimp-1. Recent studies identified STAT5 signaling as a key negative regulatory pathway of Tfh differentiation, and this inhibition was dependent on Blimp-1 expression induced by IL-2. Given that IL-2 signaling through STAT5 is also capable of constraining Th17 cell generation and IL-17 production, IL-2 seems to be an important controller of inflammation by eliminating either the Tfh cells that promote autoantibody production, or pathogenic Th17 cells, both of which contribute to autoimmune diseases. In fact, genetic deletion or blockade of IL-2 by monoclonal antibody is able to support differentiation of the Tfh [63,64] and Th17 cell population [64]. Therefore, depending on the strength of IL-2 signals, it could favor CD8 memory or Tfh and Th17 cell differentiation, when IL-2 is, respectively, present or dropped in the microenvironment [62,65]. Of note, the pathogenic Th17 cells expanding in the reduced IL-2 milieu appeared to be selectively resistant to Treg-mediated inhibition [66,67], thus strengthening chronic inflammation in autoimmunity.

## 4. IL-2 Immunotherapy in an Animal Model of Autoimmunity

Given the central role of IL-2 in Treg homeostasis, there was considerable interest in exploring its potential therapeutic effect in the mouse models of different autoimmune diseases [21,68]. Spontaneous T1D in NOD mice is the most explored experimental model of autoimmune disorders. Therefore, NOD mice represent an accepted model for testing new therapeutic approaches [69]. It has been demonstrated that low-dose IL-2 administration to NOD mice augments Treg frequency and suppressive function, thereby protecting them from chronic inflammation [5,17,18,19,20,21,22].

Numerous studies in NOD mice have shown that the effectiveness of IL-2 therapy is dependent on the stage of the disease. In particular, low-dose IL-2 could and induce reversal of the disease in pre-diabetic and new-onset NOD mice, respectively [5,21]. The mechanisms underlying T1D prevention in pre-diabetic NOD mice under treatment with low-dose IL-2 is partially associated with boosting Treg cells, especially in the pancreas. These IL-2-expanded Tregs express increased Bcl-2, CD25, and foxp3 levels, suggesting an activated state and apoptotic resistance [5]. Although low-dose IL-2 administration to recent-onset NOD mice did not lead to an increment in Treg frequencies, it was able to improve Treg suppressive function without activating pathogenic Teffs [18,21]. Furthermore, low-dose IL-2 dampened cytotoxic activity of pancreas infiltrating CD8+ T cells [21], thus providing protection from chronic inflammation and, consequently, reversed hyperglycemia. In overtly diabetic mice, IL-2 monotherapy was not sufficient for hyperglycemia reversal [70], however, IL-2 combined with sirolimus (rapamycin/RAPA) was even associated with a deleterious effect on pancreatic beta cells [18].

Studies have shown that the anti-inflammatory effect of IL-2 administration is clearly dose-dependent [18]. In contrast to beneficial low-dose IL-2 therapy (2.5 × 10^4^ IU/day), higher IL-2 doses (2.5–5 × 10^5^ IU/day) did not prevent T1D in some mice and even dramatically accelerated onset of diabetes despite local and systemic Treg improvement. In addition, high-dose IL-2 could lead to lethal toxicity [18]. This might be explained by accelerated proliferation of Teff, CD8+ T, NK, and B cells involved in T1D development in the presence of high concentrations of IL-2 [71,72]. These pathogenic high doses of IL-2-induced cells have been shown to contribute to pancreatic beta cell destruction. Therefore, it seems that IL-2 therapy might be an important driver of an imbalance between immune tolerance and destructive autoimmunity depending on its doses.

The concept of IL-2 therapy combined with other immunomodulators appeared to be optimistic in view of preliminary data, which revealed that therapy with RAPA and IL-2 could prevent autoimmune diabetes in NOD mice (2.88 × 10^3^ IU/day of rhIL-2) [73] and reduce acute graft-versus-host disease (GVHD) in an major histocompatibility complex (MHC) mismatched murine model of bone marrow transplantation (5 × 10^4^ IU/day of rhIL-2) [74]. RAPA is an inhibitor of mTOR, a component of PI3K signaling, which is predominantly expressed in Teffs, but not in Tregs; hence, RAPA administration results in a blockade of Teff proliferation. Although RAPA was beneficial when administered alone [75,76], recent studies did not confirm synergistic action of both immunotherapeutics used in combination [18], thus demonstrating limitations of IL-2 and RAPA in the therapy of T1D. In comparison with IL-2 monotherapy, RAPA + IL-2 combination failed to cure T1D due to the unexpected deleterious impact on pancreatic beta cell proliferation, glucose tolerance and metabolism in the NOD model, despite boosting Tregs. In the new-onset NOD mice previously cured by IL-2 therapy (at a dose of 2.5 × 10^4^ IU/day), RAPA could even restore diabetes, despite the low doses and short period of its administration. Hence, RAPA was shown to counteract the IL-2 impact on Treg cells, thereby breaking immune tolerance induced by low-dose IL-2 [18].

However, a short-term triple therapy regimen consisting of RAPA, agonist IL-2-related as well as antagonist IL-15-related Ig fusion proteins was shown to be suitable to effectively cure overt new-onset T1D in NOD mice [77]. Particularly, the treatment was able to suppress autoimmune destruction of the insulin-producing beta cells and convert hyperglycemia to normal levels of glucose. The durable reversal of overt T1D mice has been achieved due to combination of IL-2-mediated Treg augmentation with an antagonist to strong pro-inflammatory IL-15 cytokine, thereby leading to both permanent restoration of immune tolerance to beta cells and modification of adverse local inflammation in the pancreas. Therefore, changes in each homeostatic cytokine pathway, namely IL-2 and IL-15, may result in more profound desirable alteration in Treg/Teff cells, manifesting as permanent reversal of hyperglycemia in NOD mice [77].

The other approach with a goal to improve low-dose IL-2 therapy (3 × 10^5^ IU/day) in immune disorders is based on its combination with glucocorticoids in an animal model of GVHD [78]. Glucocorticoid (GC) treatment with dexamethasone (Dex) at a low dose of 5 mg/kg/day has been reported to selectively increase IL-2-mediated Treg *in vivo* expansion [79], thereby suppressing the development of experimental autoimmune encephalitis (EAE). Given that Dex action is dependent on inhibition of cytokine signaling, including IL-2, and Tregs express higher levels of GC receptors (GRs), CD25, and anti-apoptotic Bcl-2, these regulatory T cells are more resistant to Dex-mediated apoptosis, simultaneously sustaining higher susceptibility to IL-2-mediated activation than Teffs [80]. Moreover, it has been demonstrated that the IL-2 impact on Tregs is also expressed in selective protection from Dex-induced cell death, thus strengthening the role in immune tolerance for IL-2, especially under GC treatment. Based on the fact that Dex-treated Tregs express higher levels of cytotoxic T lymphocyte antigen-4 (CTLA-4) [81], which play a role in the inhibitory action of Tregs, all those studies strengthen the possibility of a combined strategy of autoimmune disease therapy with Dex and IL-2, depending on synergistic anti-inflammatory and immunosuppressive effects of both therapeutics. Although IL-2 could induce signaling through IL-2R and augment expansion of both Tregs and Teffs, the addition of Dex might selectively protect Tregs from apoptosis when they are activated in the IL-2 environment. In consequence, Dex + IL-2 therapy might contribute to selective expansion of desirable Tregs *in vivo*, as was seen in a murine model of GVHD after allogeneic lymphocyte transplantation [78]. These results open a new area in immune therapeutic approaches in the treatment of autoimmune diseases, especially as they are commonly cured with GCs to induce beneficial systemic immunosuppression.

Although low-dose IL-2 has an implicit potency to prevent and/or treat human autoimmune diseases, some questions still remain uncertain. First, Treg action could influence not only autoimmunity, but all immune responses, including beneficial effector responses. Second, maintenance of the proper Treg/Teff balance for immune homeostasis as well as establishing the conditions for permanent release of IL-2 to maintain a systemic Treg increase might generate some difficulties. Until recently, the latter purpose was achieved by long-term repeated systemic administration of IL-2, thereby boosting the risk of unwanted pleiotropic and systemic activity of IL-2. Yet, a very recent study has shown that sustained stimulation and local expansion of Tregs, achieved by using recombinant adeno-associated virus (rAAV) vectors expressing IL-2 at low doses, was able to improve autoimmunity without impairing immune responses to infection, vaccination, cancer development, and pregnancy [82,83]. Recent studies have also shown that rAAV-IL-2 injection resulted in achievement of different levels of IL-2 to boost tissue-resident Tregs, while avoiding the potential toxic effects of systemic IL-2 [21,57,82]. Recombinant adeno-associated virus (rAAV) is a non-integrative vector used for gene delivery to localize IL-2 long-term expression to the islets of NOD mice [68]. It is noteworthy that beta-cell-specific ectopic IL-2 did not affect conventional islet-infiltrating Teff, NK, and B cells [57]. Recombinant AAV-mediated IL-2 expanded islet-resident Tregs, thus effectively suppressing ongoing beta cell autoimmunity at late preclinical stages and prevented the onset of long-term diabetes. The study by Johnson *et al.* [57] emphasized the differential effect of beta-cell-specific IL-2 on islet-resident Tregs and Teffs. This is in line with the notion that Tregs are more sensitive to IL-2 than Teffs regarding CD25 expression [28]. Importantly, augmented tissue-resident Tregs were characterized by phenotypic changes towards an increased CD62L+/CD62L− ratio, which strengthened islet Tregs pool suppressive activity and effectively inhibited ongoing beta-cell disruption by Teffs.

All these studies emphasized the relevance of IL-2 dosing and possible interactions with other immunomodulatory drugs, which alone could improve the course of autoimmune disease. Finally, the differential effects of IL-2 therapy either alone or in combination at different stages of the disease should have been taken into consideration, when translated into clinical trials in autoimmune patients.

## 5. Low-Dose IL-2-Based Clinical Trials in Humans

Although cytokine responses in CD4+ T cells were demonstrated to influence the Treg/Teff ratio during autoimmune disease progression and therapy, the use of these indices as biomarkers of disease status seems to be problematic in some cases. Systemic autoimmunity, such as SLE or RA, is usually associated with significant changes in Treg and Teff numbers, which become more significant in disease flares. In contrast, T1D is an organ-specific autoimmune disorder where changes in cytokines and the Treg/Teff ratio in PB have not been observed with disease progression but did occur when immune therapeutics were used [84]. The central role of Tregs in immune regulation and homeostasis strengthen the rationale for using Treg-based therapy to cure various immune dysfunctions. Very recently, there were reported the results of the first clinical trials in patients with an immune disease, namely hepatitis C virus (HCV)-induced vasculitis [25], GVHD [23,24], or T1D [9,26], with a goal to induce Treg numbers and/or function with low-dose IL-2.

The first clinical use of IL-2, but at high doses of about 6–7.2 × 10^5^ IU/kg/dose every 8 h, was aimed at augmenting the immune responses against cancer. It is an immunotherapy of metastatic renal cell carcinoma and melanoma approved by the Food and Drug Administration (FDA) in 1992. IL-2 therapy has also undergone unsuccessful clinical trials for patients with HIV/AIDS [85]. High-dose IL-2 therapy of cancers has been somewhat disappointing as only 5%–10% of patients have a long-lasting complete remission. Combination therapy with IL-2 and vaccination with a melanoma peptide vaccine and the addition of adoptive cell transfer approaches significantly improved its effectiveness [86]. A limitation of high-dose IL-2 therapy in cancers is IL-2-related development of life-threatening toxicity and its short life in systemic delivery. Although an attempt to lower the dose resulted in limited undesirable side effects, it greatly dampened the therapeutic anti-tumor efficacy due to immunosuppressive Treg cell expansion, which appeared to be detrimental in cancers [87]. The notion that Treg expansion correlated with lower IL-2 doses provided new therapeutic perspectives for tolerance induction desirable in autoimmunity. However, since activated Teffs also express CD25, a high dose of IL-2 might stimulate not only Tregs, but also off-target cells. Therefore, inappropriate dosing and administration of IL-2 creates a risk of untoward immune response, including activation of self-reactive Teffs. In some cases, the dose of administered IL-2 might be too low to induce any meaningful immune response. Therefore, defining the “low-dose” range of IL-2 capable of correcting the imbalance between immune tolerance and autoimmunity appears to be essential for the treatment of autoimmune disorders.

The results of discussed clinical trials including the type of autoimmune disease, the dose and frequency of IL-2 administration, the low-dose IL-2-induced immunological changes, and the clinical outcomes are summarized in Table 1. A phase I dose-escalation trial of IL-2 in 28 patients with chronic GVHD after stem cell transplantation demonstrated beneficial effects of low-dose IL-2 by inducing selective expansion of functional Tregs and clinical improvement [23]. IL-2 was administered subcutaneously (sq) at 0.3, 1, or 3 × 10^6^ IU/m^2^ daily for 8 weeks, of which 1 × 10^6^ IU/m^2^ was defined as the maximally tolerated dose. Good clinical response, defined as at least stabilization of the disease, was observed in as many as 23 individuals (82%). In general, IL-2 therapy was well tolerated, and adverse events included arthralgia, fever, malaise, and injection side effects dependent on the relatively higher dose. The immunological changes were expressed as rapid and significant up-regulation of circulating Tregs from levels below that of healthy subjects. Other measures included eosinophilia and NK cell increase corresponding with the therapy duration. Neither Teff (CD4+), CD8, B cells nor NKT cell alterations were seen in the course of IL-2 administration. Of note, no patients enrolled in the trial experienced GVHD progression [23].

**Table 1:** The results of cited clinical trials.

Type of Autoimmune Disease	Dose (×10^6^ IU/day) and Frequency of IL-2 Administration (sq)	Cumulative Dose of IL-2 (×10^6^ IU)	Immunologic Changes	Clinical Outcome
chronic GVHD [23] ^#^ *n* = 28	0.3, 1, or 3/m^2^ × 8 weeks	100.2 *	Increase—Tregs, eosinophils, NK cells; No changes—Teffs, CD8, B cells, NKT cells	23/28 PR + SD
chronic GVHD [24] *n* = 14	0.3 or 1–1.5 × 8 weeks	16.8 or 56.0–84.0	Increase—pSTAT5 in Tregs, Tregs, serum IL2; Decrease—pSTAT5 in Teff, serum IL7 and IL-15	7/14 PR + SD
HCV-induced vasculitis [25] *n* = 10	1.5 × 5 day plus 3 courses of 3 × 5 day at weeks 3, 6, and 9	52.5	Increase—Tregs, NK cells (CD56^bright^); Decrease—B cells (marginal-zone)	8/10 PR 2/10 NR
type 1 diabetes [9] *n* = 9	4.5 × 10^6^ IU three times a week for 1 month plus RAPA 2–4 mg/day for 3 months	54.0	Increase—Tregs, eosinophils, NK cells (CD56^bright^); Decrease—neutrophils; No changes—NKT	9/9 C-peptide decrease
type 1 diabetes [26] ^#^ *n* = 9	0.33, 1, or 3 × 5 day	1.65, 5, or 15	Dose-dependent increase—Tregs, NK, Teffs; Dose-dependent decrease—B cells	24/24 no changes of C‑peptide levels

*n*: number of patients; ^#^: a dose-escalation trial; *: for a mean body surface area of 1.79; PR: partial remission; SD: stabilization of the disease; NR: no response.

The same researchers extended the study and examined the role of IL-2 administration in the homeostasis of Tregs and Teffs [24]. They examined 14 patients with refractory chronic GVHD out of those enrolled in a phase I clinical trial described above [23]. The patients received daily low-dose IL-2 (0.3, 1, or 1.5 × 10^6^ IU/day) for 8 weeks followed by a 4-week rest period without IL-2. Shortly after the study began, they observed higher levels of pSTAT5 in Teffs than Tregs in patients with chronic GVHD without IL-2 administration compared to rapidly reversed signaling imbalance between Treg and Teff in those with low-dose IL-2 therapy. Consequently, patients receiving low-dose IL-2 exhibited increased thymic generation and proliferation of functional Tregs with compromised susceptibility to apoptosis, while IL-2 had a minimal effect on the Teff population [24]. These results are in line with the notion that IL-2 has an essential role in Treg development [88] and survival [89], and administration of IL-2 at physiological doses is able to restore Treg homeostasis, thus promoting immune tolerance.

Cryoglobulinemic vasculitis is an autoimmune disorder associated with chronic hepatitis C virus (HCV) infection, and appears to be the other clinical setting marked by decreased Treg numbers. Saadoun *et al.* [25] recently reported the results of a phase I/IIa clinical trial aimed at a 4% increase of the proportion of Treg cells with low-dose IL-2 in patients with HCV-induced vasculitis. Ten HCV individuals with refractory to conventional and/or rituximab therapy vasculitis received one course of IL-2 administered sq at 1.5 × 10^6^ IU/day for 5 days followed by a 9-day rest period, with additional courses of 3 × 10^6^ IU/day for 5 days at weeks 3, 6, and 9. Similarly to the trials in GVHD, the treatment with IL-2 led to clinical improvement of vasculitis in 8 out of 10 patients. Unwanted side effects were seen only at the highest doses of IL-2, and manifested as myalgia, fatigue, flu-like syndrome, and injection site reactions. Generally, low-dose IL-2 therapy was very well tolerated, and progressively induced an expansion of Tregs with potent suppressive activity, leading to achievement of the pre-specified absolute 4% increment as the end point in as many as 80% of the HCV-related vasculitis patients at the fourth treatment course. Worthy of note, one patient maintained this 4% Treg increase even though IL-2 therapy had been stopped [25]. This clinical study confirmed the correlation of clinical improvement with boosting functional Tregs [39], and showed that low-dose IL-2 exhibited an intrinsic capacity for Treg recovery, thus improving an autoimmune condition such as HCV-induced vasculitis.

While low-dose IL-2 can safely expand/activate circulating Tregs, leading to clinical benefits in patients with GVHD or autoimmune HCV vasculitis, it was necessary to establish whether a therapeutic response may be achieved in the context of the alterations in the IL-2/IL-2R signaling pathway, which could suggest potential resistance of T1D patients to low-dose IL-2. Therefore, in this clinical setting, an optimal IL-2 dose might not be predictable and it was probable that adjuvant therapeutics would be needed to improve the outcome, as observed in NOD mice [73]. The first phase I clinical trial performed in adult T1D subjects using IL-2 at low doses investigated combined therapy with sirolimus (RAPA) [9]. As mentioned above, RAPA is an inhibitor of cell cycle progression and cytokine signal transduction preferentially in Teff cells, mainly Th1 and Th17, with a very weak or no impact on Tregs [45,46,47]; an exception was one very small study showing the beneficial impact of low doses of RAPA in monotherapy on refitting Treg suppressive function in long-term T1D subjects [76]. Therefore, the concept of a combined therapeutic strategy including RAPA with the ability to block Teff proliferation in the presence of IL-2, a crucial Treg growth factor, in diabetic patients seemed to be rational. Nine patients received orally 2–4 mg of RAPA/day for 3 months and 4.5 × 10^6^ IU/day of IL-2 three times a week for 1 month [9]. Although the Treg increase paralleled IL-2 treatment, the Treg response to IL-2 was maintained for a period of time after therapy and was observed in the follow-up studies. This may suggest that combined therapy corrects the baseline deficit in IL-2 responsiveness usually seen in T1D individuals as long as one year after treatment. However, despite desirable Treg boosting, RAPA + IL-2 combined therapy led to a transient accelerated fall in insulin C-peptide levels, suggesting exacerbated beta-cell autoimmunity manifested as worsening of islet beta-cell function. A possible explanation for the clinical and metabolic compromise seen in this trial is IL-2-mediated increase in numbers of NK cells, eosinophils, and Teff cells, despite the fact that they appeared to be untouched regarding their activation state. Nevertheless, these results emphasize the need to ascertain the unique properties of IL-2-responsive islet beta cells in T1D patients, which are a very heterogeneous population. Furthermore, the transient impact of IL-2 on the percentages of Tregs suggests the need for persistent and possibly higher levels of IL-2. It also highlights the importance of dosing and timing to minimize adverse effects of IL-2 [9].

The second phase I/II dose-finding clinical trial, conducted in a similar population with long-standing T1D, was aimed to establish the optimum dose of IL-2 (ranged from 0.33 × 10^6^ to 3 × 10^6^ IU/day) for induction of Tregs with a 5-day course in 24 patients [26]. All doses of IL-2 used in this study resulted in transient up-regulation of Tregs, accompanied by NK cell expansion and more frequent mild to moderate side effects at the higher doses of IL-2. Of note, the lack of RAPA seemed to be beneficial in T1D as no deleterious impact on beta-cell function was observed under IL-2 treatment alone, irrespectively of dose, confirming its toxic effect on beta cells [90,91,92]. RAPA was also shown to increase peripheral insulin resistance [90] or to abrogate the IL-2-induced cure of T1D in NOD mice [18]. The other explanation for unchanged glucose metabolism may be related to significantly lower cumulative doses of IL-2 used in this clinical study [26]. In the light of these findings, RAPA should not be recommended in T1D treatment in combination with IL-2 due to its negative effects on insulin C-peptide and glucose metabolism. Doses of IL-2 for use in the range 0.33–1 × 10^6^ IU/day seem to be suitable and optimal for induction of Tregs without reaching a lower limit of effectiveness.

In the light of the above clinical trials, one should note that determination of the dose range and frequency of IL-2 administration appropriate for the treatment of autoimmune disease is still a significant challenge. It is worth mentioning that in the clinical data discussed, single doses administered for the treatment of autoimmune disease were about 10–150 times lower than those used in the cancer IL-2 therapy, where cytokine was administered intravenously at doses of 6–7.2 × 10^5^ IU/kg/dose (over 15 min) every 8 h for a maximum of 15 doses, which constitutes about 42–50 × 10^6^ IU per single dose and 630–750 × 10^6^ IU as a cumulative dose (calculations assume mean body weight 70 kg). In striking contrast are the doses of IL-2 used in therapy of autoimmune diseases; IL-2 single doses ranged from 0.33 × 10^6^ to 4.5 × 10^6^ IU [9,23,24,25,26], and the cumulative dose range of IL-2 was 1.65 × 10^6^ to 100.2 × 10^6^ IU [9,23,24,25,26]. Although at the highest doses of IL-2 used (3–4.5 × 10^6^ IU/day) the Tregs enhancement was more pronounced and durable, a trend toward both NK and Teff cells’ expansion, as well as more frequent side effects lowering the therapy effectiveness, was clearly seen [9,26]. Therefore, up-to-date clinical data allow us to conclude that so-called “low-dose” IL-2 appropriately administered in the treatment of autoimmune diseases should not surpass 3 × 10^6^ IU/day, and the cumulative dose should not exceed more than 50–100 × 10^6^ IU for a course of therapy. In fact, a dose-finding clinical trial clearly showed that among different doses of IL-2 used, the lower range seems potentially non-toxic and sufficient regarding the beneficial effect on Treg boosting, and encouraged further efforts toward down-regulation of the IL-2 dose. In fact, there is an ongoing clinical study in children with recent-onset T1D assessing IL-2 at the lowest doses with its repeated administration over a longer period than those tested to date, aimed at defining the lowest sufficient dose of IL-2 and establishing kinetics of Treg enhancement, allowing suitable adjustment of its administration schedule [26].

## 6. Future Perspectives for Use of IL-2 Therapy in Autoimmune Diseases

There is an increasing body of evidence that genetic variations could be a relevant factor influencing clinical response to immunotherapeutics used [92,93]. Actually, it has been demonstrated that a significant proportion of autoimmune patients fail to respond adequately to GCs therapy, and this is, in part, a consequence of functional polymorphisms in genes encoding cytokines involved in inflammatory responses, such as IL-10 or tumor necrosis factor-alpha (TNF-alpha) [92]. In particular, a good response to GCs has been attributed to the carriage of high IL-10/low TNF-alpha genotype [92,93]. Further exploration of the association of functional IL-2 gene polymorphisms with both susceptibility to autoimmune diseases and GC therapy responses under combination with IL-2 would provide valuable clinical information.

Recently, new efforts have also been proposed including the construction of modified mutant versions of IL-2 preferentially binding the trimeric IL-2R. This procedure augments the selectivity of a modified form of IL-2 and allows one to avoid the interaction between therapeutic IL-2 and off-target cells bearing intermediate-affinity receptors. A mutant IL-2 form has been demonstrated to exhibit a few thousand-fold greater selectivity to T cells expressing the trimeric IL-2R complex than wild-type IL-2 [94]. This therapeutic approach was originally tested in the tumor environment of advanced melanoma and renal cancer patients in a phase I trial [94].

Also, an improvement of IL-2 half-life in the circulation and, in consequence, a decrement in the required dose of IL-2 is another effort with potential for clinical usage in autoimmunity. To achieve this effect, additional modifications are needed including the formation of complexes consisting of IL-2 and anti-IL-2 monoclonal antibody. Such a therapeutic strategy has already been proposed in a mouse model of allergic airway disease, a clinical setting associated with decreased number/function of Tregs [19], thereby leading to Treg-selective expansion and clinical improvement. Utilization of different monoclonal antibodies (MoAbs) conjugated with IL-2 enables one to target specific cells depending on their IL-2R affinity. The use of MoAbs to IL-2 appears to open new perspectives in the therapeutic approaches in autoimmunity, similarly to those used in cancers, because it could allow inflammation-site-specific administration of IL-2 at low doses. Site-specific IL-2 selective delivery might combine a local activity of IL-2 with its reduced systemic and pleiotropic toxicity. The antibody-cytokine fusion proteins, named immunocytokines, examined recently in animal models of cancers [95], could allow systemic infusion of low-dose IL-2 and targeted delivery to inflamed tissue, such as pancreatic beta-cells. It is an important challenge, since IL-2 acts at the crossroads of effector responses, immune tolerance, and immunotherapy [96], and functional relevance of IL-2 targeting is particularly necessary during immunosuppressive therapy of autoimmune diseases.

## 7. Conclusions

All preclinical and clinical studies discussed emphasize the potential therapeutic benefit of low-dose IL-2 therapy of autoimmune disorders. However, some difficulties in reaching and maintaining the balance between efficacy and the unwanted pleiotropic and systemic activity of IL-2 still exist and require further clinical studies to optimize the dose, timing, and schedule of IL-2 administration to minimize adverse effects of IL-2 and to limit activation of off-target cell types.
